# Sirt1: An Increasingly Interesting Molecule with a Potential Role in Bone Metabolism and Osteoporosis

**DOI:** 10.3390/biom14080970

**Published:** 2024-08-08

**Authors:** Yi Chen, Hefang Xiao, Zirui Liu, Fei Teng, Ao Yang, Bin Geng, Xiaoyun Sheng, Yayi Xia

**Affiliations:** 1 Department of Orthopedics, Lanzhou University Second Hospital, Lanzhou 730030, China; chenyi2021@lzu.edu.cn (Y.C.); 120220901471@lzu.edu.cn (H.X.); l1993070501@126.com (Z.L.); lzu_1909@126.com (F.T.); ya12021090111@126.com (A.Y.); ldeygb2008@126.com (B.G.); 2 Orthopedic Clinical Medical Research Center and Intelligent Orthopedic Industry Technology Center of Gansu Province, Lanzhou 730030, China; 3 The Second School of Clinical Medical, Lanzhou University, Lanzhou 730030, China

**Keywords:** SIRT1, bone metabolism, osteoporosis, therapeutic interventions

## Abstract

Osteoporosis (OP) is a common metabolic bone disease characterized by low bone mass, decreased bone mineral density, and degradation of bone tissue microarchitecture. However, our understanding of the mechanisms of bone remodeling and factors affecting bone mass remains incomplete. Sirtuin1 (SIRT1) is a nicotinamide adenine dinucleotide-dependent deacetylase that regulates a variety of cellular metabolisms, including inflammation, tumorigenesis, and bone metabolism. Recent studies have emphasized the important role of SIRT1 in bone homeostasis. This article reviews the role of SIRT1 in bone metabolism and OP and also discusses therapeutic strategies and future research directions for targeting SIRT1.

## 1. Introduction

Osteoporosis (OP) is a common metabolic bone disease that is characterized by low bone mass, decreased bone mineral density, and degradation of bone tissue microarchitecture, leading to increased bone fragility and a higher risk of osteoporotic fractures [1,2,3,4,5]. The pathogenesis of osteoporosis involves an imbalance between bone resorption and bone formation, influenced by hormonal changes, age, nutritional deficiencies, and genetic factors [6,7]. The interaction between osteoblasts and osteoclasts plays a very important role in bone homeostasis, keeping bone in a relatively stable environment. When osteoblasts are activated, they can express and secrete the receptor activator of nuclear factor-κB ligand (RANKL), which promotes the differentiation of osteoclast precursors into mature osteoclasts. On the contrary, when bone resorption leads to bone structure damage, osteoclasts could secrete insulin-like growth factor to promote osteogenic differentiation [8]. However, abnormal bone remodeling affects bone mass, and when osteoblasts producing calcified organic extracellular matrix are less active, bone resorption increases, osteoclasts break down the extracellular matrix, or a combination of these factors may lead to bone loss. Osteoporosis includes primary osteoporosis and secondary osteoporosis. Primary osteoporosis is subdivided into postmenopausal osteoporosis and senile osteoporosis [9]. Secondary osteoporosis can be caused by medications, prolonged immobilization, genetic predispositions, endocrine disorders, chronic kidney disease, hematological disorders, inflammatory arthropathies, nutritional and gastrointestinal disorders, and connective tissue disorders [10,11,12,13,14,15,16,17]. Understanding the mechanisms of bone remodeling and the factors that influence bone mass is essential for developing effective strategies for the prevention and treatment of osteoporosis.

Sirtuin 1 (SIRT1) is a nicotinamide adenine dinucleotide (NAD+)-dependent deacetylase that deacetylates histones and non-histone proteins [18,19,20,21]. Originally identified as a regulator of lifespan in yeast, SIRT1 was subsequently found to be involved in the regulation of oxidative stress, aging, apoptosis, DNA repair, endocrine, immune response, tumorigenesis, stress, inflammation, metabolic processes, and the regulation of a variety of physiological functions [22,23,24,25,26,27,28].

Many molecules have been found to possess the ability to regulate bone homeostasis, but the complex and adaptive body microenvironment may interfere with this role. SIRT1 has been suggested to be a potential bone homeostasis regulator gene, which plays a role in differentiation, viability, and the bone vascular system of bone marrow-derived mesenchymal stromal cells (BMSCs) and bone-forming cells [27,29]. SIRT1 affects several pathways involved in bone metabolism, including the regulation of osteoblast and osteoclast activity, thereby maintaining bone health [30]. A study has shown that SIRT1 is a positive regulator of bone mass, and its deficiency can lead to decreased bone mass and increased bone marrow fat [31]. SIRT1 has been shown to inhibit the Nuclear Factor Kappa B(NF-κB) signaling pathway, which is a key pathway in the inflammatory response to maintain normal bone reconstruction [27]. Clinical evidence suggests that reduced SIRT1 activity is associated with an increased risk of osteoporotic fractures [32]. Therefore, this review focuses on reviewing the role of SIRT1 in bone metabolism and its impact on osteoporosis, as well as exploring the potential for therapeutic interventions targeting SIRT1.

## 2. Bone Formation and the Pathogenesis of Osteoporosis

### 2.1. Bone Turnover and Formation

Bone formation is a highly coordinated process involving the differentiation and interaction of multiple cell types in the bone marrow microenvironment, including BMSCs, osteogenic precursor cells, adipocytes, osteoblasts, osteoclasts, bone marrow macrophages (BMMs), and osteocytes, each of which plays a key role in maintaining bone homeostasis and facilitating bone remodeling [6,33,34,35,36,37,38,39]. BMSCs are multipotent cells capable of differentiating into various cell types, including osteoblasts, adipocytes, and chondrocytes [35]. BMSCs are reservoirs of osteogenic precursor cells [35]. Osteogenic precursor cells proliferate and mature into osteoblasts after receiving osteogenic signals and are responsible for bone matrix synthesis and mineralization [35]. Some osteoblasts are embedded in the mineralized matrix due to the bone formation process [40]. Once embedded, these osteoblasts differentiate into osteocytes. Osteocytes are crucial because they act as mechanosensors, detecting mechanical strain within the bone [40]. This sensing ability allows them to regulate bone remodeling by signaling to other bone cells, ensuring bone strength and integrity are maintained in response to mechanical stress. Bone marrow adipocytes derived from the same pool of BMSCs secrete factors that inhibit osteoblast differentiation, affecting the overall osteogenic capacity of BMSCs, a balance that is particularly important in conditions such as aging and osteoporosis [41]. Osteoclasts are derived from BMMs and are responsible for bone resorption, degrading mineralized bone matrix, and forming resorption cavities filled with new bone matrix synthesized by osteoblasts.

Bone conversion is a tightly regulated process involving the coordinated activity of osteoclasts and osteoblasts to ensure continuous bone repair and the maintenance of bone strength [42]. OP is caused by an imbalance between these processes, characterized by increased osteoclast activity and overall bone loss [43]. Cytokines play a key role in the control of bone remodeling, with the receptor activator of nuclear factor-κB (RANK)/RANKL/osteoprotegerin (OPG) signaling systems being particularly important. RANKL produced by osteoblasts binds to the receptor RANK on osteoclasts, inducing their differentiation and activation [6]. OPG acts as a decoy receptor for RANKL and inhibits this interaction, preventing osteoclastogenesis. An imbalance in the RANK/RANKL/OPG system and elevated levels of RANKL lead to increased osteoclast activity and bone loss [6]. RANKL release is regulated by a variety of cytokines and hormones, particularly parathyroid hormone (PTH) and estrogen. PTH stimulates osteoclast activity, whereas estrogen reduces osteoclast activity through the RANK/RANKL/OPG pathway [44].

**Figure 1:**
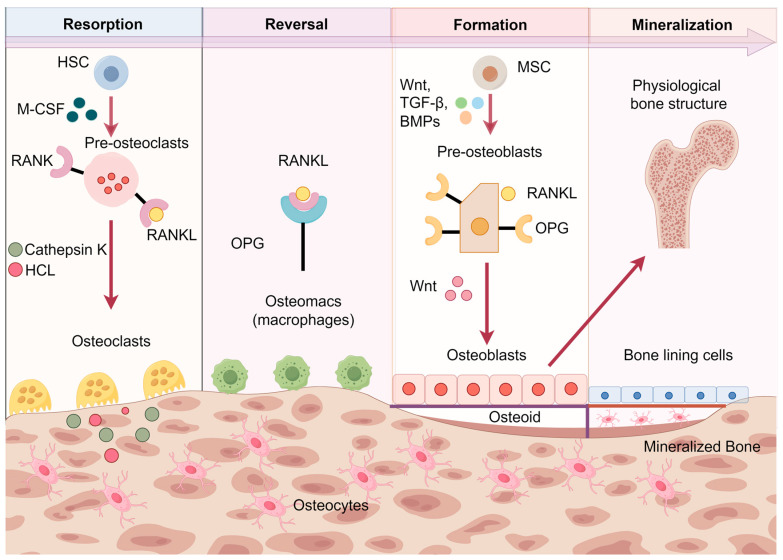
Bone remodeling cycle under physiological and pathological conditions. (1) The resorption phase, in which osteoclast precursors are induced by RANKL and M-CSF to differentiate into mature multinucleated osteoclasts. In this stage, mature osteoclasts with characteristic folded edges resorb bone by secreting Cathepsin K, H^+^, and Cl^−^ in the confined zone, and then detach from the bone surface and apoptose [6]. (2) The reversal stage, in which osteoblasts are differentiated and recruited to the resorption site in response to Wnt, BMP, and TGF-β. (3) The formative stage, in which osteoclasts form a new organic bone matrix. (4) Eventually, mineralization occurs. In conditions such as lack of estrogen or inflammation, other immune cells provide RANKL, leading to enhanced osteoclast activity and increased bone resorption [44]. HSC, hematopoietic stem cell; M-CSF, macrophage colony-stimulating factor; RANK, receptor activator of nuclear factor-κB; RANKL, receptor activator of nuclear factor-κB ligand; OPG, osteoprotegerin; MSC, mesenchymal stromal cell; BMPs, bone morphogenetic proteins; TGF-β, transforming growth factor-β. Created by Figdraw.com (https://www.figdraw.com).

The bone remodeling process consists of four successive phases: (1) the resorption phase, controlled by RANKL and macrophage colony-stimulating factor (M-CSF), which induces the differentiation of hematopoietic stem cell-derived osteoclast precursors into mature multinucleated osteoclasts. At this stage, mature osteoclasts with characteristic folded edges resorb bone by secreting histone K, H^+^, and Cl^−^ in the seal zone, and then detach from the bone surface and undergo apoptosis [6]. (2) In the reversal stage, in the presence of Wnt, bone morphogenetic proteins (BMPs) and transforming growth factor-β (TGF-β) are present, and osteoblasts of mesenchymal origin differentiate and are recruited to the site of resorption. (3) Formative phase, where osteoblasts form a new organic bone matrix. (4) Eventually undergo mineralization. Under conditions such as lack of estrogen or inflammation, other immune cells provide RANKL, leading to enhanced osteoclast activity and increased bone resorption, ultimately leading to osteoporosis [44] (Figure 1).

### 2.2. Pathogenesis of Osteoporosis

Osteoporosis is mainly caused by an imbalance between bone resorption and bone formation, leading to a decrease in bone mass and the degradation of bone tissue microstructure [45]. This imbalance is influenced by a variety of factors, including estrogen, aging, oxidative stress, mechanical stimuli, inflammation, obesity, and metabolic disorders [46,47]. Estrogen deficiency, especially in postmenopausal women, is a major contributor to increased bone resorption by osteoclasts and decreased bone formation by osteoblasts [33]. Estrogen maintains bone density by regulating osteoclast apoptosis and the expression of RANKL and OPG, which are key mediators of osteoclastogenesis [48]. Decreasing estrogen levels lead to up-regulation of RANKL and down-regulation of OPG, which enhance osteoclast activity and bone resorption, which in turn cause postmenopausal osteoporosis [49,50]. Senile osteoporosis (SOP), is characterized by reduced bone formation [51]. Studies have shown that SOP-containing BMSCs undergo senescence, which is associated with telomere shortening, oxidative stress, and genetic and epigenetic regulation [52]. In addition, studies have found lower bone mineral density in older populations of chronic smokers, most notably in the hip [40].

Glucocorticoid-induced osteoporosis (GIO) is another common form of secondary osteoporosis in which chronic glucocorticoid use leads to suppressed bone formation and increased bone resorption [53]. Glucocorticoids inhibit osteoblast proliferation and differentiation by suppressing the Wnt signaling pathway, which is essential for osteoblast function and survival [54,55]. In addition, glucocorticoids enhance osteoclastogenesis by up-regulating RANKL expression and reducing OPG production [53]. This dual effect on bone remodeling may result in significant bone loss and an increased fracture risk [53]. In addition, glucocorticoids increase oxidative stress by promoting reactive oxygen species (ROS) production [56]. The increased ROS levels lead to enhanced osteoclast activity by stimulating RANKL expression, and they impair osteoblast function by inducing apoptosis and inhibiting differentiation [56].

Oxidative stress and inflammation play a key role in the pathogenesis of osteoporosis. ROS generated by cellular metabolism and external stressors can induce osteoblast apoptosis and promote osteoclast differentiation, leading to bone loss [56]. Chronic inflammation, characterized by elevated levels of pro-inflammatory cytokines such as TNF-α, IL-1, and IL-6, further enhances osteoclast activity and inhibits osteoblast function [57,58]. The interaction between oxidative stress and inflammation creates a vicious cycle that accelerates bone resorption and impairs bone formation, leading to the progression of osteoporosis [40].

In addition, recent studies have highlighted the impact of metabolic factors on bone health. High blood glucose levels and advanced glycosylation end products (AGEs) have been shown to induce apoptosis in osteoblasts and disrupt the expression of osteosclerostin and RANKL, which are essential for bone remodeling [59,60]. Bone marrow adipose tissue (BMAT), which increases with age and obesity, negatively affects bone metabolism by secreting adipokines and inflammatory cytokines that promote osteoclastogenesis and inhibit osteoblast activity [61]. These findings highlight the importance of considering the complex interactions between hormones, aging, oxidative stress, inflammation, obesity, and metabolism in the diagnosis and treatment of osteoporosis.

Understanding and studying the pathogenesis of osteoporosis is important for clinical management. Although existing treatments (e.g., bisphosphonates, estrogen replacement therapy, etc.) are capable of slowing down bone loss to a certain extent, their side effects, safety concerns for long-term use, and poor efficacy in certain patient populations suggest that there is still an unmet clinical need for the prevention and treatment of osteoporosis [62]. These drugs are often accompanied by a range of side effects, such as bisphosphonates that may lead to gastrointestinal discomfort, osteonecrosis of the jaw, and atypical femur fractures, while estrogen replacement therapy may increase the risk of cardiovascular disease and breast cancer [63,64]. Additionally, the safety of long-term use of these drugs has also raised widespread concern, especially in patients with chronic diseases that require long-term management [65]. Therefore, new therapeutic strategies and targets are urgently needed to improve treatment efficacy and minimize side effects. Ideal treatments should be efficient and safe, providing a better balance between slowing bone loss and promoting bone production while minimizing the risk of side effects. The exploration of new therapeutic targets and the development of new drugs will not only help to meet existing clinical needs, but also provide new directions for personalized treatment of osteoporosis, which will ultimately improve patients’ quality of life and treatment outcomes.

## 3. SIRT Family

Sirtuins are conserved NAD+-dependent deacetylases, a family of longevity proteins initially identified as lifespan regulators in yeast that regulate key signaling pathways in both prokaryotes and eukaryotes and are involved in many biological processes [66]. The family of sirtuins includes seven members (SIRT1-SIRT7); each member has unique and overlapping functions and can be classified into four types according to their structural similarities [67]. SIRT1, SIRT2, and SIRT3 belong to type I, SIRT4 belongs to type II, SIRT5 belongs to type III, and SIRT6 and SIRT7 belong to subtypes IVa and IVb of type IV, respectively [68,69].The Sirtuins family are NAD+-dependent deacetylases and ADP-ribosyltransferases, which play key roles in inflammation, apoptosis, cellular metabolism, stress tolerance, and aging, and have been considered potential therapeutic targets for a wide range of diseases such as cardiovascular diseases, endocrine disorders, neoplasms, and respiratory diseases [70,71,72]. SIRT1 is the most studied member and has been shown to regulate a variety of pathways associated with aging and metabolic homeostasis, including glucose and lipid metabolism, mitochondrial function, and inflammation [73,74,75]. SIRT1 achieves these effects by deacetylating key transcription factors and co-regulators, such as p53, NF-κB, and PGC-1α [69,76,77].

The deacetylation activity of SIRT1 requires the assistance of NAD+, which is also a cofactor involved in DNA damage repair. SIRT2 is predominantly found in the cytoplasm, is involved in the regulation of the cell cycle and cytoskeletal organization, and has been shown to deacetylate microtubule proteins and histones, affecting cell division and gene expression [78,79]. SIRT3, SIRT4, and SIRT5 are mitochondrial longevity proteins that regulate mitochondrial function and metabolism [69]. SIRT3 enhances ATP production and reduces oxidative stress by deacetylating and activating enzymes involved in the tricarboxylic acid cycle and oxidative phosphorylation [80,81]. SIRT4 counteracts the effects of caloric restriction on insulin secretion by inhibiting glutamate dehydrogenase [77]. SIRT5 has been shown to regulate the mitochondrial lysine succinylome, affecting metabolic networks and oxidative stress responses [82]. SIRT6 and SIRT7 are found predominantly in the nucleus and play roles in DNA repair, genome stability, and transcriptional regulation [79,83]. SIRT6 deacetylates histone H3, thereby stabilizing DNA-dependent protein kinases on chromatin to repair DNA double-strand breaks [79]. It also regulates glucose homeostasis by modulating the expression of glycolytic genes [68]. SIRT7 has been identified as a histone desuccinylase associated with chromatin compression and genome stability, further emphasizing the importance of long-lived proteins in maintaining genome integrity [83] (Table 1).

The involvement of the Sirtuins family in various cellular processes makes them key players in health and disease. SIRT1, in particular, is thought to be associated with the beneficial effects of caloric restriction and has been shown to extend the lifespan of a variety of organisms [84,85]. The role of the Sirtuins family in metabolic homeostasis and their protective effects against oxidative stress and inflammation have made them promising targets for the treatment of metabolic disorders and diseases associated with aging, such as osteoporosis and type 2 diabetes [18,80,86,87].

**Table 1:** Location, functions, and enzyme activities of different sirtuins.

Sirtuin	Subcellular Localization	Enzyme Activity	Functions	References
SIRT1	Nucleus and cytoplasm	Deacetylase	Cell survival, metabolism regulation, oxidative stress response, inflammatin, mitochondrial biogenesis, life span regulation	[68,69,74,78,87,88,89,90]
SIRT2	Nucleus and cytoplasm	Deacetylase	Neurodegeneration, cell cycle regulation, tumor suppression/promotion	[91,92,93,94,95]
SIRT3	Nucleus, cytoplasm and mitochondria	Deacetylase	Protection against oxidative stress, tumor suppression, mitochondrial metabolism	[96,97,98,99,100]
SIRT4	Mitochondria	Deacetylase ADP-ribosylase Lipoamidase	Tumor suppression, amino acid catabolism, mitochondrial metabolism	[101,102,103,104]
SIRT5	Mitochondria	Deacetylase Desuccinylase Demalonylase	Apoptosis, urea cycle, amino acid metabolism, fatty acid metabolism	[68,69,85,105,106,107]
SIRT6	Nucleus	Deacetylase ADP-ribosylase	DNA repair, genome stability, glucose and lipid metabolism, inflammation	[108,109,110,111]
SIRT7	Nucleus	Deacetylase	Cell cycle regulation, ribosome biogenesis, rRNA transcription	[112,113,114,115]

## 4. The Physiological Functions of SIRT1

SIRT1 is considered a longevity factor because it extends lifespan by resisting oxidative stress during aging [116]. SIRT1 regulates the activity of a variety of key transcription factors and proteins through deacetylation and affects their stability, localization and interactions with other molecules [18]. For example, by deacetylating the tumor suppressor protein p53, SIRT1 leads to a reduction in p53-mediated apoptosis and enhances cell survival under stress conditions [117]. This deacetylation activity also extends to other substrates, such as NF-κB, leading to suppression of inflammatory gene expression, thus playing an important role in reducing inflammation and cellular senescence [71,118]. In addition, SIRT1 interacts with histone H1 to promote selective heterochromatin formation, thereby affecting gene silencing and chromatin structure [119]. SIRT1 epigenetically silences target proteins such as p53, Forkhead box O (FoxOs), and β- catenin, and other target proteins are silenced epigenetically [120,121,122,123].

In terms of metabolic regulation, SIRT1 has an important role in glucose and lipid metabolism. SIRT1 enhances mitochondrial biogenesis and oxidative metabolism by deacetylating activated peroxisome proliferator-activated receptor-gamma coactivator 1-alpha (PGC-1α) [23,76]. This regulation is essential for the maintenance of energy homeostasis, especially during fasting and calorie restriction [23,76]. SIRT1 also regulates the activity of various metabolic transcription factors, including peroxisome proliferator-activated receptor γ (PPARγ), liver X receptor (LXR), and farnesoid X receptor (FXR), which in turn affect lipid metabolism and insulin sensitivity, demonstrating its role in controlling obesity and related metabolic disorders [124]. In adipose tissue, SIRT1 inhibits PPARγ activity, promotes lipolysis, and reduces fat storage, which is essential for the prevention of obesity and metabolic syndrome [124]. In the liver, SIRT1 enhances gluconeogenesis and inhibits glycolysis through deacetylation and activation of PGC-1α and FOXO1, aligning energy production with metabolic demand [125,126]. In terms of liver function, SIRT1 also regulates hepatic gene expression, further highlighting its role in metabolic regulation [127]. In addition, SIRT1 regulates growth and development by interacting with the insulin-like growth factor signaling pathway [128]. SIRT1’s role in metabolic regulation also includes involvement in insulin resistance and glucose tolerance. SIRT1 regulates insulin sensitivity through neural networks by reversing insulin resistance through the activation of duodenal SIRT1 [129].

In bone metabolism, SIRT1 regulates bone homeostasis by modulating the activity of osteoblasts and osteoclasts, which are responsible for bone formation and resorption, respectively [30]. SIRT1 reduces bone resorption by inhibiting NF-κB signaling in osteoclasts and promotes osteoblast differentiation and function through the deacetylation of the key osteogenic transcription factor, runt-related transcription factor 2 (RUNX2) [27,130]. In addition, SIRT1 enhances the antioxidant defense of osteoblasts by interacting with Forkhead box O3A (FOXO3A) and protecting these cells from oxidative stress-induced apoptosis, thereby sustaining bone formation [131]. The dual regulatory roles of SIRT1 in bone formation and bone resorption highlight its potential as a therapeutic target for the treatment of osteoporosis and other bone-related diseases [132,133].

The versatility of SIRT1 extends to a variety of other physiological processes, including embryogenesis, germ cell production, and neuroprotection. SIRT1 plays a critical role in embryonic development and reproduction, and mice deficient in SIRT1 exhibit developmental defects and impaired germ cell production [134,135]. SIRT1 regulates testosterone biosynthesis in Leydig cells by modulating autophagy, demonstrating its role in reproductive health [136]. In addition, SIRT1 regulates the expression of cartilage-specific genes involved in maintaining cartilage integrity [137]. In the central nervous system, SIRT1 protects neurons from neurodegenerative diseases by regulating pathways involved in oxidative stress and inflammation [67]. The neuroprotective function of SIRT1 has been linked to its ability to regulate metabolic transcription factors and improve insulin sensitivity, which is essential for neuronal health [126]. In addition to its role in neuroprotection, SIRT1 affects vascular health by regulating endothelial function and preventing vascular senescence and atherosclerosis. Studies have shown that SIRT1 plays a key role in maintaining vascular smooth muscle cell function and reducing vascular inflammation, thereby preventing age-related vascular disease [25,138]. In addition, SIRT1 is involved in immune regulation, affecting the function of various immune cells and playing a role in diseases such as systemic lupus erythematosus [139]. SIRT1 affects the stability and function of regulatory T cells, which play a key role in controlling graft-versus-host disease [24].

**Figure 2:**
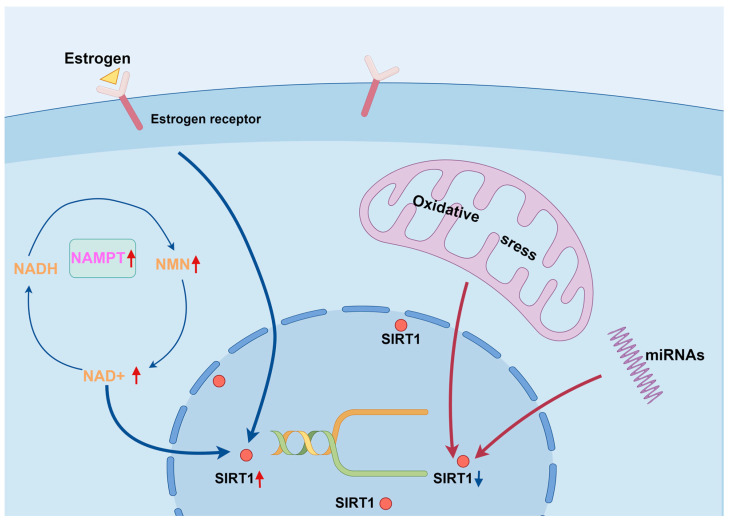
Model of the upstream pathways targeting SIRT1 in control of bone homeostasis. SIRT1 is a NAD+-dependent enzyme, NMN is a precursor substance for NAD+, and NAMPT is a reliable biomarker of osteogenic differentiation [141]. The AMPK pathway can activate SIRT1 through the AMPK/NAMPT pathway, increasing the NAD+/NADH ratio, which is mainly involved in energy metabolism [142,143,144,145,146]. MiRNAs can bind to the mRNA of SIRT1, thereby inhibiting its translation process [147]. Excess ROS can oxidize cysteine residues that modify SIRT1, thereby inhibiting SIRT1 [149]. Estrogen can up-regulate SIRT1 expression [30]. NMN, nicotinamide mononucleotide; NAMPT, nicotinamide phosphoribosyltransferase; NAD+, nicotinamide adenine dinucleotide; NADH, nicotinamide adenine dinucleotide hydride; miRNAs, microRNAs. Created by Figdraw.com (https://www.figdraw.com).

It has been found that NAD+ can promote osteogenic differentiation of BMSCs in a SIRT1-dependent manner [29]. Similarly, nicotinamide mononucleotide (NMN), a precursor substance of NAD+, can promote osteogenic differentiation of MSCs by activating SIRT1 [140]. In addition, Li et al. [141] found that nicotinamide phosphoribosyltransferase (NAMPT) is a reliable biomarker of osteogenic differentiation. The AMPK pathway can activate SIRT1 through the AMPK/NAMPT pathway, increasing the NAD+/nicotinamide adenine dinucleotide hydride (NADH) ratio, which is mainly involved in energy metabolism [142,143,144,145,146]. Therefore, activation of SIRT1 via NMN or nicotinamide riboside (precursors of SIRT1) to increase the NAD+/NADH ratio has become a strategy for the treatment of aging and X-ray induced osteoporosis [140] (Figure 2). Recently, by studying the post-translational regulation of SIRT1, some researchers have found that microRNAs (miRNAs) can bind to the mRNA of SIRT1 and thus inhibit the translation process [147]. SIRT1 is known to be a NAD +-dependent enzyme that is closely related to oxidative stress [148]. Excess ROS can inhibit SIRT1 by oxidatively modifying the cysteine residues of SIRT1 [149]. Yoon et al. [150] found that the antioxidant factor nuclear factor E2-related factor 2 up-regulates the expression of SIRT1, which resists oxidative stress by inhibiting p53. Elbaz et al. [30] found that estrogen can inhibit PPARγ in ovariectomized mice by inhibiting PPARγ to enhance SIRT1 expression and reduce adipogenesis (Figure 2).

## 5. The Role of SIRT1 in Osteoblasts, Osteoclasts, Bone Marrow Mesenchymal Cells, and Osteocytes

### 5.1. SIRT1 and Osteoblasts

The physiological function of osteoblasts is essential for bone formation, and SIRT1 maintains bone health by regulating functional aspects of osteoblasts. Huang et al. [151] showed that SIRT1 overexpression protects mouse osteoblasts from TNF-α-induced injury by inhibiting the NF-κB signaling pathway. Edwards et al. [27] further demonstrated this protective mechanism and found that SIRT1 maintains physiological bone remodeling by inhibiting NF-κB signaling, thereby preventing excessive bone resorption and promoting osteoblast survival. In addition, SIRT1 also plays a key role in regulating osteoblast proliferation and differentiation through interactions with various transcription factors. Iyer et al. [122] revealed that SIRT1 promotes cortical bone formation by preventing the capture of β-catenin proteins in osteoblast precursors by the FoxO transcription factor. This is supported by Abed et al. [152], who found that the SIRT1 activator resveratrol enhanced the phenotypic characteristics and activity of osteoblasts in osteoarthritis, suggesting that SIRT1 activation can improve osteoblast function under pathological conditions. SIRT1 also plays a key role in protecting osteoblasts from apoptosis, especially under stressful conditions. For example, Deng et al. [153] found that SIRT1 protects osteoblasts from particle-induced inflammatory response and apoptosis in sterile prosthesis loosening. Similarly, Zhou et al. [154] found that overexpression of SIRT1 prevented apoptosis of osteoblasts under hypoxic conditions, highlighting the role of SIRT1 in enhancing cell survival under unfavorable conditions. Yao et al. [155] further demonstrated the anti-apoptotic effect of SIRT1, showing that SIRT1 up-regulation through the FoxO1/β-catenin pathway inhibited H_2_O_2_-induced osteoblast apoptosis.

**Figure 3:**
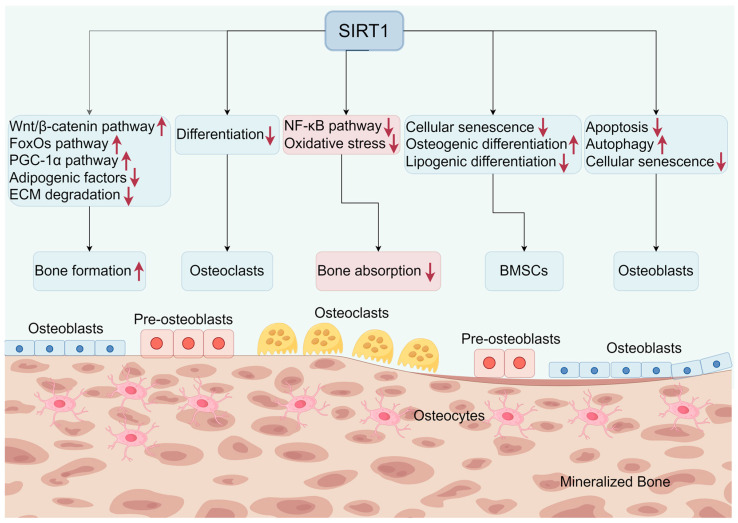
SIRT1 maintains bone homeostasis by targeting a series of downstream signaling pathways. SIRT1 regulates bone formation and bone resorption through osteoblasts, osteoclasts, and BMSCs. Wnt/β-catenin, wingless-related integration site/Beta-catenin; FoxOs, forkhead box O; PGC-1α, peroxisome proliferator-activated receptor-gamma coactivator 1-alpha; ECM, extracellular matrix; NF-κB, nuclear factor kappa B; BMSCs, bone marrow-derived mesenchymal stromal cells. Created by Figdraw.com (https://www.figdraw.com).

Furthermore, the role of SIRT1 in osteoblasts extends to metabolic regulation. Bai et al. [156] demonstrated that NADPH oxidase isozymes play a role in glucocorticoid-induced apoptosis in preosteoblasts, and that SIRT1 attenuates these effects by regulating oxidative stress. A recent study by Jin et al. [157] revealed that SIRT1 regulates osteoblast glycolysis through glutamic-oxaloacetic transaminase 1 (GOT1) to maintain bone homeostasis, suggesting that the metabolic regulatory function of SIRT1 is critical for osteoblast activity and bone health. Gu et al. [158] found that SIRT1 exerts a protective effect under fluoride-exposed conditions, suppressing p53-dependent apoptosis by modulating p21 in MC3T3-E1 cells, further emphasizing the protective effect of SIRT1 on osteoblasts under toxic conditions. In addition, Qu et al. [159] found that SIRT1 affected osteogenic proliferation and differentiation of MC3T3-E1 cells by regulating miR-132-3p, suggesting that SIRT1 not only protects osteoblasts but also enhances their proliferation and differentiation. Recent studies have also highlighted the role of SIRT1 in osteoblast autophagy. Yang et al. [160] demonstrated SIRT1-mediated resveratrol-regulated autophagy in osteoporotic rat osteoblasts, suggesting that SIRT1 activation enhances autophagic processes in osteoblasts, thereby promoting cell survival and function. In addition, Zhou et al. [161] found that SIRT1 reduces osteoblast senescence via superoxide dismutase 2 (SOD2) acetylation and mitochondrial dysfunction. Domazetovic et al. [162] found that blueberry juice antioxidants were involved in protecting osteogenic activity from oxidative stress via SIRT1 and improved long-term activation of mineralization processes in human osteoblast-like SaOS-2 cells, suggesting that dietary antioxidants enhance osteoblast function through SIRT1 activation. Zuo et al. [163] revealed that 17β-estradiol improves osteoblast function through the SIRT1/NF-κB/MMP-8 pathway, suggesting that SIRT1 mediates the beneficial effects of estrogen on osteoblasts. Wang et al. [164] demonstrated that the up-regulation of SIRT1 induced by 17β-estradiol (17β-E2) could promote autophagy via the AMPK-mTOR pathway and inhibit apoptosis via FOXO3A activation in osteoblasts. Recent studies have explored the molecular mechanisms underlying the protective effects of post-translational modifications of SIRT1 on osteoblasts. Cai et al. [165] demonstrated that the SIRT1 Asn346 glycan chain protects osteoblasts in response to stress by promoting collagen deacetylation. SIRT1 exerts a multifaceted role in regulating osteoblast function, including anti-apoptosis, anti-inflammatory response, anti-aging, promotion of differentiation and proliferation, enhancement of autophagy, and metabolic regulation (Figure 3).

### 5.2. SIRT1 and Osteoclasts

The role of SIRT1 in regulating osteoclastogenesis and osteoblast function has been extensively studied [166]. Osteoclasts are responsible for bone resorption, a key process in bone remodeling, and their overactivity can lead to pathological conditions such as osteoporosis [167]. Kim et al. [168] revealed that SIRT1 regulates high mobility group protein 1 (HMGB1)-induced osteoclastogenic cytokines in human periodontal ligament cells, emphasizing the importance of SIRT1 in controlling the inflammatory response that drives osteoclast differentiation. In addition, Shakibaei et al. [169] showed that the resveratrol-mediated interaction of SIRT1 with p300 regulates the activation of NF-κB signaling by NF-κB receptor activator (RANKL), which inhibits osteoclastogenesis in bone-derived cells. SIRT1 is essential for maintaining physiological bone remodeling through inhibition of the NF-κB pathway. Edwards et al. [27] observed that SIRT1 maintains physiological bone remodeling, prevents excessive bone resorption, and promotes bone health by inhibiting NF-κB signaling. This inhibitory effect was further supported by Gurt et al. [170], who found that the SIRT1 activators, SRT2183 and SRT3025, inhibit RANKL-induced osteoclastogenesis in bone marrow-derived macrophages and down-regulate SIRT3 in SIRT1-deficient cells, emphasizing the role of SIRT1 in regulating osteoclast activity through multiple pathways.

In addition, SIRT1 plays an important role in the metabolic regulation of osteoclasts. Park et al. [171] showed that cilostazol inhibited RANKL-induced osteoclast differentiation through SIRT1-induced RANK inhibition, suggesting that SIRT1 activation can reduce osteoclastogenesis through metabolic pathways. Kim et al. [172] found that SIRT1 inhibited mouse osteoclast progenitor cell proliferation and reduced osteoclastogenesis, and conversely, SIRT1 deletion in osteoclast progenitor cells increased osteoclast number and bone resorption. They also discovered that the anti-osteoclastogenic effect of SIRT1 is mediated by FoxO and caused by impaired mitochondrial activity. Furthermore, Qu et al. [173] found that SIRT1 inhibited high glucose and palmitate-induced osteoclast differentiation by deacetylating p66Shc, suggesting a role for SIRT1 in regulating osteoclast metabolism under high glucose conditions.

The role of SIRT1 in oxidative stress regulation is also critical for osteoclast function. Zhang et al. [174] found that granule-induced down-regulation of SIRT1 promotes osteoclastogenesis and osteolysis through endoplasmic reticulum stress regulation, suggesting that SIRT1 protects against osteoclast differentiation induced by oxidative stress. Yan et al. [175] further showed that SIRT1 directly inhibits osteoclastogenesis by inhibiting ROS generation and transient receptor potential vanilloid 1 (TRPV1) channel activation under the mediation of TNF-α. Ye et al. [176] found that apoptotic extracellular vesicles mitigated Pg-LPS-induced inflammatory responses in macrophages through the AMPK/SIRT1/NF-κB pathway and inhibited osteoclast formation, suggesting that SIRT1 activation can regulate macrophage responses and osteoclastogenesis under inflammatory conditions. SIRT1 regulates osteoclast function through inhibition of inflammatory and oxidative stress pathways, modulation of metabolic responses, and direct effects on osteoclast differentiation and activity (Figure 3).

### 5.3. SIRT1 and Bone Marrow Mesenchymal Cells

SIRT1 plays an important role in regulating the fate of BMSCs, influencing their differentiation into osteoblasts or adipocytes [31]. It has been shown that activation of SIRT1 promotes osteogenesis while inhibiting adipogenesis in BMSCs. This dual role is important for the maintenance of bone homeostasis and the prevention of osteoporosis. Bäckesjö et al. [177] found that activation of SIRT1 reduced adipocyte formation during osteogenic differentiation of mesenchymal stromal cells (MSCs), highlighting the importance of SIRT1 in directing the differentiation of BMSCs towards osteogenesis. This is further supported by Tseng et al. [178], who showed that resveratrol up-regulates RUNX2 gene expression through the SIRT1/FOXO3A axis to promote osteogenesis in MSCs. The promotion of osteogenic differentiation by SIRT1 through the modulation of transcription factors is one of the key mechanisms. Shakibaei et al. [179] showed that resveratrol mediated SIRT1/RUNX2 regulation promoted osteogenic differentiation of MSCs, possibly through RUNX2 deacetylation. This finding is consistent with Simic et al. [180], who reported that SIRT1 regulates MSC differentiation through deacetylation of β-catenin, thereby enhancing osteogenic differentiation. In addition, SIRT1 plays an important role in maintaining the self-renewal and pluripotency of BMSCs. Yoon et al. [181] found that SIRT1 directly regulates SRY-Box Transcription Factor 2 (SOX2) to maintain the self-renewal and pluripotency of BMSCs. This regulatory mechanism ensures a pool of precursor cells required for osteogenic differentiation, which is essential for bone regeneration and repair. This function is particularly important in the context of senescence, as the regenerative potential of BMSCs is often affected by senescence.

Recent studies have provided additional evidence for the protective role of SIRT1 in osteogenesis. Sun et al. [182] showed that overexpression of SIRT1 in MSCs protects mice from bone loss through FOXO3A deacetylation and inhibition of oxidative stress. This protective effect is essential for maintaining the functional integrity of BMSCs under stress conditions. Hou et al. [183] found that Bergenin acts through activating SIRT1 as a novel therapeutic agent to promote osteogenesis in BMSCs, further highlighting the therapeutic potential of SIRT1 activation in enhancing osteogenic differentiation and bone regeneration. Qu et al. [184] demonstrated that in high glucose and free fatty acid microenvironments, overexpression of miR-449 inhibited osteogenic differentiation of BMSCs by suppressing the SIRT1/FRA-1 pathway, suggesting that SIRT1 is also involved in the metabolic regulation of BMSCs under stress conditions. Liu et al. [185] found that β-mercaptoethanol promotes osteogenesis in human MSCs through the SIRT1/extracellular regulated protein kinases (ERK) pathway, further clarifying the role of SIRT1 in mediating cellular responses to oxidative stress.

The role of SIRT1 in metabolic regulation also extends to its promotion of osteogenesis and reduction in adipogenesis in aged bone marrow. Song et al. [140] reported that nicotinamide mononucleotides regulate MSCs in aged bone marrow through the SIRT1 pathway to promote osteogenesis and reduce adipogenesis. The dual role of SIRT1 in regulating osteogenesis and adipogenesis is essential to preventing age-related bone loss and maintaining bone health. Wang et al. [186] further showed that SIRT1 promotes osteogenic differentiation and increases alveolar bone mass in mice through B cell-specific Moloney murine leukemia virus integration site 1 (BMI1) activation, suggesting that SIRT1 is a key regulator of bone mass and bone volume. The antioxidant properties of SIRT1 are also critical for maintaining the osteogenic potential of BMSCs. Chen et al. [187] found that melatonin restored the osteogenic potential of osteoporosis-injured BMSCs by maintaining SIRT1-mediated intracellular antioxidant properties, demonstrating the importance of SIRT1 in protecting BMSCs from oxidative damage and enhancing their osteogenic capacity.

In addition, Chen et al. [188] further elucidated that SIRT1 ameliorated age-related MSC senescence by regulating telomere protection proteins, thereby preserving the stemness and proliferative capacity of BMSCs. SIRT1 is also important in maintaining the self-renewal and pluripotency of BMSCs. Wang et al. [189] found that exogenous NAD+ delayed D-galactose-induced senescence of BMSCs via SIRT1 signaling, further highlighting the role of SIRT1 in protecting BMSCs from senescence and maintaining their regenerative potential. Kou et al. [190] reported that eldecanate prevented OVX-induced osteoporosis by inhibiting BMSC senescence through modulation of SIRT1/Nuclear Factor Erythroid 2-Related Factor 2 (Nrf2) signaling, suggesting that SIRT1 activation enhances the regenerative potential of BMSCs and prevents bone loss. Liu et al. [191] found that salvianolic acid C promotes osteoporosis through activation of the AMPK/SIRT1 pathway and osteogenic differentiation of rat BMSCs. The above studies further support the therapeutic potential of SIRT1-regulated activity of BMSCs in enhancing osteogenesis and preventing osteoporosis (Figure 3).

### 5.4. SIRT1 and Osteocytes

To date, the role of SIRT1 in osteocytes has been relatively understudied compared to its role in other bone cell types. Osteocytes are the most numerous cells in the skeleton and are critical for bone homeostasis. Embedded in the mineralized matrix, osteocytes form an extensive network of dendrites that regulate the activity of osteoblasts and osteoclasts, thereby coordinating bone remodeling [192,193,194]. The protective effect of SIRT1 on osteocytes is mainly achieved by attenuating oxidative stress, which is an important factor contributing to the apoptosis of osteocytes and subsequent bone loss [195]. Domazetovic et al. [195] showed that blueberry juice, partly via SIRT1, protects osteocytes and bone precursor cells from oxidative stress. This study highlights the importance of SIRT1 in enhancing the antioxidant defenses of osteocytes, thereby maintaining their function and survival under conditions of oxidative stress. In addition, SIRT1 regulates the expression of sclerostin, a glycoprotein produced by osteocytes that inhibits osteoblast activity and bone formation. Kim et al. [196] found that regulation of sclerostin by the SIRT1 stabilization pathway in osteocytes is essential for the maintenance of bone homeostasis. Specifically, SIRT1 stabilizes sclerostin by preventing its ubiquitination and subsequent degradation, thereby ensuring its availability for regulating osteoblast function. This regulatory mechanism highlights the complex role of SIRT1 in balancing bone formation and bone resorption by influencing osteocytes. In addition, interactions between SIRT1 and other signaling pathways in osteocytes, such as mechanosensitive pathways, are critical for the adaptive response of bone to mechanical loading, which is essential for maintaining bone strength and integrity [194,197,198,199,200,201].

The effect of SIRT1 on osteocyte metabolism is also important. As metabolically active cells, osteocytes require a finely regulated balance of energy production and expenditure to maintain their function [202,203,204,205]. Liu et al. [206] found that osteocyte TSC1 inhibited osteogenesis in mice by promoting sclerostin secretion, suggesting that metabolic regulation within osteocytes is critical for their role in bone remodeling. SIRT1, through enhancement of mitochondrial function and biogenesis, increases osteocytes’ metabolic requirements, and this metabolic support is particularly important under conditions of metabolic stress. The role of SIRT1 in the regulation of osteocyte function also extends to effects on cellular stress responses. Rawal et al. [207] highlighted the importance of SIRT1 in attenuating insulin resistance and its associated metabolic dysregulation, which may also have implications for osteocyte function. The ability of SIRT1 to enhance stress resistance and promote cellular homeostasis is critical for osteocyte longevity and function. This function is supported by the interaction of SIRT1 with other stress-responsive proteins and pathways, which together enhance osteocyte resilience under various stress conditions.

## 6. The Role of SIRT1 in the Pathogenesis of Different Types of Osteoporosis

Studies have shown that different interventions may affect the expression of SIRT proteins in human samples and that supplementation with SIRT modulators may have different effects on physiological function in different participants [208]. SIRT1 plays a key role in the pathogenesis of osteoporosis, particularly in postmenopausal women and the elderly. SIRT1 maintains bone homeostasis by regulating a variety of cellular processes, including oxidative stress, inflammation, and the differentiation of osteoblasts and osteoclasts [130,209]. Elbaz et al. [30] showed that estrogen deficiency, the main cause of postmenopausal osteoporosis, led to an increase in bone marrow adipogenesis and a decrease in the expression of SIRT1, emphasizing the key role of SIRT1 in the maintenance of bone health in the presence of hormonal changes. SIRT1 has been shown to increase bone mass by regulating osteoblast and osteoclast activity. Zhang et al. [210] found that globular C1q/tumor necrosis factor-related protein-3 (gCTRP3) inhibited ovariectomy-induced osteoporosis through activation of the AMPK/SIRT1/Nrf2 signaling pathway in mice, further supporting a role for SIRT1 in protecting against estrogen-deficiency-induced bone loss via metabolic and oxidative stress pathways. Zainabadi et al. [130] reported that SIRT1 activation enhanced bone mass and acted as a therapeutic target for osteoporosis, emphasizing the potential of SIRT1 in maintaining BMD and preventing bone loss. A role for SIRT1 was also found in senile osteoporosis. Ameen et al. [211] further elucidated the possible role of resveratrol in the treatment of age-related osteoporosis in men through activation of the FoxO1/SIRT1/RANKL/OPG pathway, suggesting complex interactions between SIRT1 and key signaling pathways of bone metabolism. Hong et al. [212] also found that atorvastatin promotes bone formation through the SIRT1-Runx2 axis in senescent apoE(^−/−^) mice, further reinforcing the role of SIRT1 in enhancing osteoblast activity and bone formation. Wen et al. [213] found that low-intensity vibration alleviated age-related bone loss, by inhibiting cellular senescence in naturally senescent rat osteoblasts, and partly by up-regulating SIRT1, emphasizing the protective role of SIRT1 in counteracting oxidative stress-induced cellular senescence.

Oxidative stress is an important factor in the pathogenesis of osteoporosis, and SIRT1 plays a key role in mitigating its effects. Kim et al. [214] showed that decreased NAD+ levels lead to the loss of osteogenic progenitor cells and bone mass, and that activation of SIRT1 counteracts these effects by enhancing resistance to oxidative stress and promoting osteogenic differentiation. SIRT1 also regulates the production of the glycoprotein sclerostin, which inhibits osteoblast activity and bone formation. Zeng et al. [215] showed that oxidative protein products exacerbated age-related bone loss through ROS-dependent down-regulation of SIRT1 and increased sclerostin expression in osteoblasts, suggesting the importance of SIRT1 in regulating osteoblast function and maintaining bone formation. Cohen-Kfir et al. [26] reported that SIRT1 acts as a repressor of Sost (encoding sclerostin), further supporting a role for SIRT1 in enhancing osteoblast activity and bone formation. In addition, SIRT1 has a protective role in glucocorticoid-induced osteoporosis. Hou et al. [216] found that ferulic acid, a natural polyphenol, protects against glucocorticoid-induced osteoporosis in neonatal rats through activation of SIRT1 and NF-κB, suggesting that activation of SIRT1 attenuates the adverse effects of glucocorticoids on bone mass. Huang et al. [217] showed that nicotinamide mononucleotide attenuated glucocorticoid-induced osteogenesis inhibition by modulating the SIRT1/PGC-1α signaling pathway, further highlighting the therapeutic potential of SIRT1 activators in glucocorticoid-induced osteoporosis. The interactions between SIRT1 and other signaling pathways also play a key role in the pathogenesis of osteoporosis. Liu et al. [218] showed that long chain non-coding RNA TRG-AS1 protects against glucocorticoid-induced osteoporosis in a rat model by regulating the miR-802-mediated CAB39/AMPK/SIRT1/NF-κB axis, highlighting the role of a complex regulatory network involving SIRT1 in bone metabolism. Xiao et al. [219] found that STK11 overexpression prevented glucocorticoid-induced osteoporosis by activating the AMPK/SIRT1/PGC1α axis, further supporting the role of SIRT1 in regulating key metabolic pathways to protect bone health.

In addition, Jin et al. [220] found that vitamin K2 inhibited long-term hyperglycemia-mediated bone loss and iron death and that vitamin K2 treatment restored bone mass and enhanced the expression of SIRT1, GPX4, and osteogenic markers in the distal femur. The specific mechanism is that vitamin K2 may ameliorate type 2 diabetic osteoporosis by inhibiting iron death through activation of the AMPK/SIRT1 signaling pathway. SIRT1 also affects epigenetic regulation of bone metabolism. Wang et al. [221] reported that METTL14 attenuated the development of osteoporosis in ovariectomized mice by up-regulating the m6A level of SIRT1 mRNA, suggesting that epigenetic modification of SIRT1 has a significant effect on bone mass. Although many studies have elucidated the positive role in bone-related diseases, there is a lack of clinical trials in this domain.

## 7. The Application of SIRT1 Agonists in Osteoporosis

In recent years, the potential of SIRT1 agonists in the treatment of osteoporosis has attracted much attention due to their ability to modulate key pathways in bone metabolism [66,130,222,223,224,225]. SIRT1 agonists such as resveratrol and the synthetic agonists SRT2104 and SRT3025 have demonstrated bone-protecting effects in preclinical and clinical studies [224,225,226]. These agonists act primarily by enhancing SIRT1 activity, which affects osteoblast and osteoclast function, reduces oxidative stress, and attenuates inflammation.

Resveratrol, a natural polyphenol found in grapes and red wine, is one of the most studied SIRT1 agonists. Feng et al. [227] reported that resveratrol exerts a protective effect on postmenopausal osteoporosis by modulating the SIRT1-NF-κB signaling pathway, decreasing inflammation, and promoting osteogenesis. In addition, Lee et al. [226] found that resveratrol supplementation enhanced bone growth in young rats and improved microarchitecture and remodeling in older rats, further supporting its potential as a treatment for osteoporosis [226]. In a clinical trial, Bo et al. [228] showed that resveratrol supplementation positively impacted bone health in patients with type 2 diabetes, demonstrating its broad applicability in metabolic bone disease. A recent study by Jiang et al. [229] demonstrated that resveratrol promotes osteogenesis in osteoporotic mice through activation of the SIRT1/FoxO1 pathway, revealing its molecular mechanism for bone protection. However, the therapeutic indications of resveratrol mainly target diabetes and cardiovascular diseases rather than bone-related diseases, and since resveratrol is not a specific agonist of SIRT1, its multiple off-target effects are a key factor to be considered. In addition, Hou et al. [183] further identified Bergenin as a novel therapeutic agent that promotes osteogenesis in bone MSCs through activation of SIRT1.

## 8. Summary and Future Perspectives

With a deeper understanding of the role of SIRT1 in bone metabolism and osteoporosis, future research is promising, and the following directions are particularly worth exploring: (1) Although the important role of SIRT1 in bone metabolism and osteoporosis has been clarified, its specific molecular mechanisms still need to be further elucidated. In addition, the specific roles of SIRT1 in different cell types and its epigenetic regulatory mechanisms are key areas for future research. (2) The role of SIRT1 in regulating the skeletal microenvironment is not yet fully understood. Future studies could focus on how SIRT1 regulates bone reconstruction and bone health by affecting microenvironmental components such as bone marrow mesenchymal stem cells, bone marrow adipocytes, and bone marrow microvessels. (3) Although studies have demonstrated the potential of SIRT1 activators (e.g., resveratrol) in the prevention and treatment of osteoporosis, systematic clinical trials are still insufficient. Future studies should focus on validating the efficacy and safety of these activators in different populations (e.g., postmenopausal women, the elderly, patients with glucocorticoid-induced osteoporosis, etc.). (4) Currently, SIRT1 lacks bone targeting, and systemic administration makes it difficult to achieve effective drug concentrations in bone tissue; future studies need more specific and selective SIRT1 activators. (5) Osteoporosis is often accompanied by other metabolic diseases, such as diabetes and cardiovascular diseases. Studying the role of SIRT1 in these complications and its interaction with osteoporosis will help to fully understand the multiple biological functions of SIRT1 and develop comprehensive treatment programs.

In summary, this review highlights the important role of SIRT1 in bone metabolism and osteoporosis and provides a theoretical basis for the potential of SIRT1 as a new therapeutic target for osteoporosis. By gaining a deeper understanding of the molecular mechanisms of SIRT1, conducting systematic clinical trials, and exploring its role in the skeletal microenvironment and complications, we are expected to develop more effective strategies for osteoporosis prevention and treatment and improve the quality of life of patients.
